# Local people’s views on the evidence-based skilled-maternal-care in Mfuwe, Zambia: a qualitative study

**DOI:** 10.1186/s12884-019-2282-y

**Published:** 2019-04-24

**Authors:** Choolwe Muzyamba

**Affiliations:** 1 UNU MERIT, Boschstraat, 246211 AX Maastricht, The Netherlands; 2 A9 Marshlands Village Box 32379, Lusaka, Zambia

**Keywords:** Evidence-based, Skilled-care, TBAs, Maternal health, SSA

## Abstract

**Background:**

There is growing demand for high quality evidence-based practice in the fight against negative maternal health outcomes in Sub-Saharan Africa (SSA). Zambia is one of the countries that has transposed this evidence-based approach by outlawing Traditional Birth Attendants (TBAs) and recommending exclusive skilled-care. There is division among scholars regarding the usefulness of this approach to maternal health in SSA in general. One strand of scholars praises the approach and the other criticizes it. However, there is still lack of evidence to legitimize either of the two positions in poor-settings. Thus the aim of this study is to fill this gap by investigating local people’s views on the evidence-based practice in the form of skilled-maternal-care in Zambia, by using Mfuwe as a case study.

**Methods:**

With the help of the Social Representation theory, Focus Group Discussions (FGDs) were conducted in Mfuwe, Zambia with 63 participants.

**Findings:**

The study shows that the evidence-based strategy (of exclusive skilled-care) led to improved quality of care in cases where it was accessible. However, not all women had access to skilled-care; thus the act of outlawing the only alternative form of care (TBAs) seemed to have been counterproductive in the context of Mfuwe. The study therefore demonstrates that incorporating TBAs rather than obscuring them may offer an opportunity for improving their potential benefits and minimizing their limitations thereby increasing access and quality of care to women of Mfuwe.

**Conclusion:**

This study illustrates that while evidence-based strategies remain useful in improving maternal care, they need to be carefully appropriated in poor settings in order to increase access and quality of care.

## Plain English summary

Strengths:

-Study investigates local people’s own understanding of the usefulness of the evidence-based approach

-It focuses on a seldom-researched subject despite its important implication on maternal health care in Zambia

Weaknesses:

-It has a small study population thereby limiting the external validity of the study.

## Background

With most of Sub-Saharan Africa (SSA) still burdened with high maternal mortality, evidence-based practice has gained popularity within maternal health care response as a way of addressing this scourge. Evidence-based practice in health care is defined as the integration of the best available clinical knowledge epitomized by the application of the most high quality intervention to a given health problem [1–3]. For example, exclusive utilization of skilled maternal care characterizes evidence-based care within maternal health response in Zambia [4]. It is considered so because it has been proven to reduce both maternal and child mortality when effectively applied [2]. As such, there is growing demand for high quality evidence-based practice in the fight against negative maternal health outcomes in SSA. Zambia, given its high maternal mortality [4, 5] has transposed this approach into national maternal health policy [6]. This means that Zambia has outlawed any form of care which does not fall under “skilled-care”; particularly, Traditional Birth Attendants (TBAs) who are considered unsafe and lacking essential skills and equipment to guarantee quality care [6].

The exclusive adoption of skilled maternal care in poor settings has attracted division among scholars. One strand of scholars praises the approach on grounds that by exclusively relying on scientifically-proven techniques such as skilled-attendants, there is increased access to quality care which reduces maternal mortality [7]. Where it has been effectively applied, evidence-based practice has been credited for improving maternal health outcomes, and it is for this reason that most scholars are recommending it in areas which are most-affected by maternal mortality. On the other hand, there is a strand of scholars who argue against the universalization of this approach. These scholars posit that exclusive focus on evidence-based practice is unhelpful in poor settings because it is ideal but unattainable [8, 9]. The approach is further criticized for giving little importance to local realities and the various ways local people handle their own maternal health burden.

Despite increased debate on this issue, not much is known about how useful the approach is to the local people it is meant to serve, especially in poor settings with high maternal mortality. Thus this study aims to fill this gap by investigating local people’s views on the evidence-based maternal care (exclusive utilization of skilled-care) in Mfuwe, Zambia. The study is conducted in Mfuwe, Zambia. Mfuwe is a rural settlement located in the South Luangwa national park in the Eastern province of Zambia. There exits only one hospital in Mfuwe (Kamoto hospital) catering for a population of over 207,000 people spread roughly around an arear of 370 km^2^. The settlement retains high HIV rates, including very high maternal and under five mortality rates in the country [5, 10]. It is for this reason that Mfuwe was selected as a case study.

## Theoretical framework

To achieve its aims, the study makes use of the Social Representations theory; which is a framework used to investigate the different ways local people make sense of a given social phenomenon [11]. This theory argues that local people while acting as a collective are able to negotiate and collectively shape their own understanding and give meaning to a social phenomenon [11]. Thus in order to understand the usefulness of a given social phenomenon, investigating people’s own characterization of that particular phenomenon is crucial. This is especially important in investigating the usefulness of health interventions in specific settings. It is for this reason that the study uses this theory heuristically to guide the process of data collection, data analysis and presentation of results. In this sense, the framework helps us to investigate and understand the usefulness of evidenced-based care from the perspective of locals.

## Methods

In order to address the aim of this study, we conducted a qualitative study in Mfuwe, Zambia in February of 2016.

### Sampling

To recruit participants, we relied on convenient and purposive sampling techniques. This method of sample selection was chosen due to the fact that our population of interest was hard to reach. Mfuwe is a sparsely-populated and very large settlement which lacks proper road network making it difficult to reach several women who are remotely located.

### Recruitment

In total we recruited sixty-three participants. All participants were women who had at least given birth once prior to the study and had utilized either skilled-care or TBAs during their pregnancy. Thirty three of them had made use of skilled-attendants in health facilities and the other thirty utilized the services of TBAs. The selected participants varied in age, marital status, educational level and occupation. The variety was good for increasing diversity of opinions expressed. Participants were selected from within Mfuwe district of Zambia. Participants who met the criteria were approached and asked to be part of the study, those who were willing and able to participate were then recruited. Table 1 shows the summary of demographic and social characteristics of the participants.

**Table 1:** Participants’ demographics

ID	Age	Highest Education Level	Employment status
Women who utilized professional care			
1	40-	No Education	Unemployed
2	30–39	Secondary Education	Unemployed
3	20–29	Secondary Education	Employed
4	20–29	Vocational Training	Unemployed
5	<20	Secondary Education	Unemployed
6	30–39	Primary Education	Unemployed
7	20–29	No education	Employed
8	20–29	Primary education	Unemployed
9	<20	Secondary Education	Unemployed
10	30–39	Primary Education	Unemployed
11	<20	Secondary Education	Unemployed
12	30–39	Primary Education	Unemployed
13	20–29	No education	Employed
14	20–29	Primary education	Unemployed
15	<20	Secondary Education	Unemployed
16	30–39	Primary Education	Unemployed
17	> 40	No Education	Employed
18	30–39	Vocational Training	Unemployed
19	20–29	No Education	Unemployed
20	20–29	Primary Education	Employed
21	20–29	No education	Employed
22	20–29	Primary education	Unemployed
23	20–29	No education	Employed
24	<20	Secondary Education	Unemployed
25	30–39	Primary Education	Unemployed
26	<20	Secondary Education	Unemployed
27	30–39	Primary Education	Unemployed
28	30–39	Primary Education	Unemployed
29	20–29	No education	Employed
30	20–29	Primary education	Unemployed
31	<20	Secondary Education	Unemployed
32	30–39	Vocational Training	Unemployed
33	20–29	No Education	Unemployed
Women who did not utilized professional care			
34	> 40	Vocational training	Unemployed
35	> 40	Vocational Training	Employed
36	<20	Primary Education	Employed
37	30–39	Primary Education	Unemployed
38	20–29	No education	Employed
39	20–29	Primary education	Unemployed
40	<20	Secondary Education	Unemployed
41	30–39	Primary Education	Unemployed
42	> 40	No Education	Employed
	30–39	Primary Education	Unemployed
43	> 40	No Education	Employed
44	30–39	Vocational Training	Unemployed
45	20–29	No Education	Unemployed
47	20–29	Primary Education	Employed
48	<20	Secondary Education	Unemployed
49	30–39	Primary Education	Unemployed
50	<20	Primary Education	Unemployed
51	<20	Secondary Education	Employed
52	30–39	Primary Education	Unemployed
53	<20	Primary Education	Unemployed
54	30–39	No education	Employed
55	20–29	Primary Education	Unemployed
56	30–39	University Education	Unemployed
57	> 40	Vocational training	Unemployed
58	30–39	Vocational training	Employed
59	20–29	Vocational Training	Employed
60	20–29	Primary Education	Unemployed
61	20–29	Vocational training	Employed
62	30–39	Vocational Training	Unemployed
63	> 40	Primary Education	Unemployed

### Data collection

Six different focus group discussions (FGDs) were conducted separately. The first five groups each comprised of ten participants and the last group had thirteen participants. Each of the FGDs contained a mix of women who had utilized skilled-care and those who utilized TBAs. FGDs were conducted in different locations and villages within Mfuwe in January and February of 2016. The FGDs lasted an average of ninety minutes. All FGDs were conducted in Chichewa (the local language) and English was used where possible. The FGDs were guided by a topic guide and in some cases we allowed for varied discussions emerging from the discussion. The FGD guide included about thirteen general questions ranging from reasons why participants chose to use skilled-care or TBAs, their experiences with these services, their views regarding how beneficial and suitable these services were, what they liked or did not like, what was lacking etc. follow up questions were also asked to ensure thorough discussions

### Analysis

The data were first translated from Chichewa to English and then analyzed in NVivo. NVivo is a software that allows researchers to evaluate, summarize and give meaning to qualitative data [12]. In NVivo, the data is evaluated and summarized around major themes, a process collectively known as thematic analysis. Thematic analysis helps to examine and describe different phenomenon by the use of emerging themes arising from the data [12]. The analysis in this study followed this approach. In this regard, similar opinions from participants were grouped together in NVivo to form various clusters of basic themes which later give rise to global themes. The summary of these results is presented in Table 2.

**Table 2:** Summary of qualitative results

Global Theme	Basic Findings	
Positive characterization of evidence based care (skilled care)	Easily handle complications	• Professionals have more knowledge and skills to handle complications than trained TBAs
		• Professionals have access to equipment and medical supplies that can deal with HIV vulnerability during pregnancy but TBAs don’t
		• As opposed to TBAs, professionals can conduct completed procedures such as caesarian births
		• Have access to life-serving medical equipment used to conduct complicated operations
	Help promote PMTCT	• Help in PMTCT
		• Help Easily conduct HIV tests
		• Help in the provision of ARVs
		• Help in conducting caesarian births
Negative characterization of evidence-based care (skilled care)	inaccessible	• Health facilities are located very far away
		• Skilled care is expensive
		• There is poor infrastructure e.g. transport system
		• Not enough health facilities
		• In some areas there is no health centers
	Takes away the only feasible option of care	• TBAs a re now outlawed
		• It has made it difficult to access TBAs
		• There is nolonger cheap and easily accessible provided psychological, emotional and economic support
		• Made it very difficult to access antenatal and postnatal care from home (home-based care)
		• Counterproductive government policy on TBAs as it Creates barriers in accessing the most vulnerable women in remote areas
	TBAs are now unregulated	• Despite being outlawed TBAs are still being used
		• There is no monitoring or regulation of TBAs
		• No chance of improving the skills of the only available form of care TBAs
		• Policy on skilled care frustrates cooperation between professionals and
		• TBAs

### Ethical clearance

Before conducting the study, we obtained written ethical clearance from two ethical clearance institutions in Zambia. a) The National Health Research Authority of Zambia, and b) the Zambian Eres-converge ethics board. We also ensured that informed written consent was sought from the participants prior to participation. Participants were also informed of their right to opt out of the interviews at any point should they feel the need to do so.

## Results

The Social Representation theory helped to demonstrate that participants had both positive and negative experiences with skilled-care. In general, they seemed to be critically aware of the several benefits of skilled-care and at the same time they highlighted the various ways exclusive focus on skilled-care denied them feasible opportunities of care. More specifically:

### Positive characterization of skilled-care

In many ways, local women praised skilled-care for being able to identify and effectively handle complications that arise during antenatal, delivery and postnatal periods. They specifically mentioned how useful technologically-advanced medical knowledge and equipment have been in identifying life-threatening illnesses. Different participants indicated how different risks such as preeclampsia, obstructed delivery and fistula were effectively handled by skilled health workers.

“*It is because of my doctor’s ability to identify and use ultrasound imaging that they saw what was wrong with my pregnancy and they were able to handle the complication. Imagine if I did not go to the hospital? What would have become of me? Some of these complications really need the attention of highly-skilled personnel”*

It was further highlighted that skilled-care was useful in identifying HIV cases and enrolling women who turned out to be HIV positive on antiretroviral therapy (ART). To further ensure Prevention of Mother to Child Transmission (PMTCT), medical officers provided ART and conducted caesarean births. Skilled attendants also ensured that treatment continued after birth including provision of useful information on breastfeeding and nutrition.

*“It’s only when I went to the hospital that I discovered my HIV status and the doctors enrolled me on treatment. They also advised me to go the hospital regularly for checkups and in the process performed a caesarean procedure on me. So in a way I would say the doctors were really helpful in ensuring that my child is free from HIV and for that I am grateful”.*

### Negative characterization of professional care

While acknowledging the benefits of skilled-care, some women remained critical of the government’s policy of exclusive skilled-care which was in most cases unavailable to them. They lamented about how most health facilities were remotely-located and inaccessible. Although they preferred skilled-care, it did not seem to be a realistic option for them.

*“They say we need to deliver only in hospitals. But have you seen how far the nearest hospital is? Do you know how much it cost to get there? And how much the services are in those hospitals? Most of us here do not earn any money, but they are asking us to go to hospitals without providing any transportation. It is ridiculous. If they really cared, they would have constructed hospitals close to us.”*

Some women also spoke against the dominance of the “skilled-attendance” approach which they accused of discounting and obscuring any feasible locally-available options. They wondered why authorities insisted on skilled-care while ignoring locally-available alternatives such as TBAs who for generations had carried the burden of maternal care amidst inefficient health systems. While highlighting the many limitations that TBAs presented (such as lack of equipment and skills to handle complications) they wondered why government had outlawed the only readily available option of care in their context.

*“I have always wondered why there is so much concentration on skilled-attendance…. Do you know how this concentration has obscured all our culture and the possibilities of relying on our own people like TBAs. Do you hear any talk about TBAs today? It does not really add up you know. TBAs have traditionally been helping us over the years, they are good.. of course they lack some skills and what not, but they are what we have. How do you tell us not to use them. And the only option you have given is a hospital located 11km away. What happens if my labor starts in the night, without transport?”*

Participants in the study were also of the view that the act of focusing exclusive on evidence-based care not only denied most women access to care, but also made it impossible to maximize benefits of TBAs and minimize their costs. They stated that because of the exclusive focus on skilled-care and outlawing of TBAs, there was little attention on regulating TBAs (who they still continued to use albeit illegally). According to the participants, the banning of TBAs did not eradicate their presence on the market, it just took them ‘underground’, away from the regulation and control of authorities. Thus some women pointed out that because of lack of regulation, the only available option had become even riskier.

*“Imagine if the government had given as much attention to TBAs; we could have seen a situation where access to care is increased and TBAs could be accorded opportunities to improve their skills… the current situation is a lose-lose situation both for the women and the government”*

## Discussion

With the help of the Social Representation theory, this study has documented in different ways the benefits and limitations of evidence-based care in a poor setting like Mfuwe. In terms of its benefits, the study showed that skilled-care was useful in identifying and handling complications which arise during pregnancy and childbirth. This inherently helped to reduce both maternal and child mortality. The study comes to the same conclusion as other studies from southern African region which indicate that skilled-care is indeed an important and necessary approach in arresting most of the maternal health challenges faced in resource-poor settings like Mfuwe, Zambia [7, 9, 13].

While it is true that skilled-care care was useful in handling complications, it however remained inaccessible to several women in Mfuwe [10, 14]. This means that there were several women who were unable to enjoy the benefits of this approach. More specifically, in agreement with other studies [6, 14, 15], we posit that insisting on exclusive skilled-care when it is inaccessible to many may be counterproductive if no useful alternative is provided to the marginalized [14]. In the case of Mfuwe, it seems legitimate to argue that the well-intended evidence-based approach failed to connect with the realities of this remote poor setting which is characterized by various structural barriers. This ultimately speaks to the fact that when context is ignored, evidence-based practice may sometimes fail to yield its intended benefits.

Further, the action to outlaw TBAs (the only alternative form of care), coupled with the inaccessible skilled-care did nothing to eradicate TBAs from the market. Out of necessity, women continued to covertly utilize them as they were the only feasible option. Our respondents including other studies from Mfuwe [10, 14] confirm that despite the ban, women continue to utilize services of TBAs out of necessity. TBAs continue to operate illegally making any possibilities of improving their potential benefits and minimizing their limitations impossible. This is because in this way, TBAs cannot be monitored, regulated nor given further training. This may have worsened the quality of services offered by TBAs much to the detriment of local women [6, 10]. This observation is in line with what other scholars have continued to show elsewhere within SSA [15–19]. It therefore seems legitimate to postulate as others have done elsewhere that any attempt to exclusively focus on skilled-care in such a setting is realistically and practically unrewarding [20, 21]. Thus without careful inspection, some evidence-based strategies may appear coherent and logical on face value, but may in actual fact remain completely blunt instruments in complex poor settings like Mfuwe. The continued quest to achieve these unattainable goals (exclusive skilled-care) in the case of Mfuwe seemed to have made the perfect the enemy of the good. This observation is consistent with other studies from within the region which all show that unless it is contextualized, evidence-based care fails to achieve its aims in poor settings [3, 22–25].

Our findings demonstrate that in order to promote maternal care in areas like Mfuwe, there is a need to create space for engagement between evidence-based interventions and local strategies. It was clear from our study that for some women, skilled-care is not an option, and it is for this reason that they resort to TBAs. While it is true that TBAs present several limitations, they, at the same time remain the only option for some women. Thus incorporating them rather than obscuring them may offer an opportunity for improving their potential benefits and minimizing their limitations. TBAs can also be useful in handling non-technical tasks in order to complement the efforts of the skilled-attendants. Studies coming from rural parts of Zambia indicate that when existing community resources are used, there is great potential for scaling up much-needed care [26, 27]. For example, the Safe Motherhood Action Group (SMAG) initiative demonstrated that through incorporating community workers including TBAs, access to health-enhancing maternal care in rural parts of Zambia increased [28]. Similarly, other studies from the region have shown that including unskilled community workers in the handling of non-technical tasks increased access to care and also improved maternal outcomes [22, 29, 30]. This approach also allowed the unskilled community workers to receive appropriate training and supervision in order to improve their service-delivery. In the same way, TBAs in Mfuwe can be included in the line of care to complement skilled-care. In this regard, they can be trained, regulated and assigned appropriate roles. This could lead to greater coverage of quality care in Mfuwe in a manner that is regulated, feasible, contextually-suitable and beneficial. Currently, the existing policy on maternal care seems to be focused on an ideal form of care (evidence-based) which given the structural limitations in Mfuwe, seems unattainable [14]. Thus there seems to be more to benefit from the inclusion of TBAs in the official line of care rather than discounting and ignoring them [14].

### Limitations

In this study, we note the following limitation. Due to logistical and financial constraints, the findings of this study are based only on the views of participants who were located in only one of the ten provinces of Zambia and were all selected via convenient and purposive sampling techniques. This means that the study was prone to selection bias meaning that the results may not be very representative. Further, due to the large number of participants in FGDs, participants might have lacked adequate space to fully express themselves. Although this study from Zambia was adequate and relevant in giving insights into the usefulness of evidence-based (skilled-care) care to women living in rural-settings, in order to solidify our findings, there is need for more studies on this subject.

## Conclusion

This study illustrates that while evidence-based strategies remain useful in improving maternal care, they need to be carefully implemented in given context. The study showed that skilled care was useful in improving quality of care to those who could access it. However, such care remains elusive to some women in poor settings. Thus banning of other forms of care and exclusively focusing on skilled-care denied women the only feasible alternative means of care, and in some cases, gave rise to unregulated and unmonitored operation of TBAs much to the detriment of women. This means that policy on maternal health should take into account the contrasting outcomes and characterization of popular maternal health initiatives in different context. Uncritical adherence and mechanical-rule-following of established standards (in this case skilled-care) may sometimes deny the women it is meant to serve the much-needed care. This study therefore demonstrates that in order to improve the quality of maternal care in Mfuwe, TBAs must be included in the line of care in order to minimize their shortcomings and maximize their potential benefits. This approach also allows them to be trained, regulated and assigned appropriate roles.

## Abbreviations

AIDS: Acquired Immune Deficiency Syndrome
ART: Antiretroviral Treatment
HIV: Human Immunodeficiency Virus
PMTCT: prevention of Mother to Child Transmission
SSA: Sub-Saharan Africa
TBA: Traditional Birth Attendants
WHO: World health Organization

## References

[1] M. G. Titler, “the evidence for evidence-based practice implementation”. *in Patient Safety and Quality: An Evidence-Based Handbook for Nurses,* 2008.

[2] Forland F, Rohwer AC, Klatser P, Boer K, Mayanja-Kizza H. “Strengthening evidence-based healthcare in Africa”. Br Med. 2013;108(10):606–8. 10.1136/eb-2012-101143 23416418

[3] Cornish F. “Thinking methods – when the demand for ‘evidence’ is unscientific: an example from HIV/AIDS”. LSE blogs. 2015;6(11):1–2.

[4] Stekelenburg J, Kyanamina S, Mukelabai IW, V. Roosmalen J (2004). Waiting too long: low use of maternal health services in Kalabo, Zambia. Tropical Med Int Health.

[5] ZDH, “Zambia Demographic Health Survey,” (2013). Central Statistical Office [Zambia].

[6] Cheelo C, Nzala S, Zulu JM (2016). Banning traditional birth attendants from conducting deliveries: experiences and effects of the ban in a rural district of Kazungula in Zambia. BMC Pregnancy Childbirth.

[7] Harrison K. A. (2011). Are traditional birth attendants good for improving maternal and perinatal health? No. BMJ.

[8] Greenhalgh T., Howick J., Maskrey N. (2014). Evidence based medicine: a movement in crisis?. BMJ.

[9] McMichael C, Waters E, Volmink J (2005). Evidence-based public health: what does it offer developing countries?. J Public Health.

[10] Muzyamba C, Groot W, Tomini S, Pavlova M (2018). Community mobilization and maternal Care of Women Living with HIV in poor settings: the case of Mfuwe, Zambia. BMC Health Services.

[11] Moscovici S, Flick U (1998). The history and actuality of social representations. The psychology of the social.

[12] Braun V, Clarke V (2006). Using thematic analysis in psychology. Qualitative research in psycholog.

[13] Ministry-of-Health (2013). Roadmap for accelerating reduction of Maternal, Newborn and child mortality, 2013-2016.

[14] Muzyamba C, Groot W, Pavlova M, Tomin S (2017). The usefulness of traditional birth attendants to women living with HIV in resource-poor settings: the case of Mfuwe, Zambia. BMC Tropical Medicine and Health.

[15] Ana J. (2011). Are traditional birth attendants good for improving maternal and perinatal health? Yes. BMJ.

[16] Crowe M, Utley A, Costello A, Pagel C (2012). How many births in sub-Saharan Africa and South Asia will not be attended by a skilled birth attendant between 2011 and 2015?. BMC Pregnancy and Childbirth.

[17] Campbell Oona MR, Graham Wendy J (2006). Strategies for reducing maternal mortality: getting on with what works. The Lancet.

[18] S. Timmermans and M. Berg, “The gold standard: the challenge of evidence-based medicine and standardization in health care,” Temple University Press*,* 2003.

[19] Schneider H, Hlophe H, van Rensburg D (2008). Community health workers and the response to HIV/AIDS in South Africa: tensions and prospects. Health Policy & Planning.

[20] Sialubanje C, Massar K, Hamer DH, Ruiter RA (2015). Reasons for home delivery and use of traditional birth attendants in rural Zambia: a qualitative study. BMC journal of Pregnancy and Childbirth.

[21] Marcos Y, Phelps B, G B (2012). Community strategies that improve care and retention along the prevention of mother-to-child transmission of HIV cascade: a review. J Int AIDS Soc.

[22] Campbell C, Scott K, Nhamo M, Nyamukapa C, Madanhire C, Skovdal M, Sherr L, Gregson S (2013). Social capital and HIV competent communities: the role of community groups in managing HIV/AIDS in rural Zimbabwe. AIDS Care.

[23] C. Catling, N. Medley, M. Foureur, C. Ryan and N. T. A. H. Leap, “Group versus conventional antenatal care for pregnant women,” Cochrane*,* vol. 2, 2015. 10.1002/14651858.CD007622.pub3 PMC6465187 25922865

[24] Dehne K, Wacker J, Cowley J (1995). Training birth attendants in the Sahel. World Health Forum.

[25] Balogun M, Odeyemi K (2010). Knowledge and practice of prevention of mother-to-child transmission of HIV among traditional birth attendants in Lagos state, Nigeria. The Pan African Medical Journal.

[26] Ensor Tim, Green Cathy, Quigley Paula, Badru Abdul Razak, Kaluba Dynes, Kureya Tendayi (2013). Mobilizing communities to improve maternal health: results of an intervention in rural Zambia. Bulletin of the World Health Organization.

[27] Green C, Soyoola M, Surridge M, Kaluba D (2014). A training approach for community maternal health volunteers that builds sustainable capacity. Dev Pract.

[28] Quigley P, Green C, M S, Kureya T, Barber C, Mubuyaeta K (2018). Empowering women and communities to promote universal health coverage in rural Zambia. Dev Pract.

[29] S. Bergstrom and E. Goodburn, “The role of traditional birth attendants in the reduction of maternal mortality,” *Safe*How many births in sub-Saharan Africa and South Asia will not be attended by a skilled birth attendant between 2011 and 2015? *motherhood strategies: a review of the evidence,* p. 451, 2001.

[30] Dambisya Y, Matinhure S. Policy and programmatic implications of task shifting in Uganda: a case study. BMC Health Services. 2012;12(61). 10.1186/1472-6963-12-61 PMC3352120 22409869

